# Decreased B and T lymphocyte attenuator in Behcet’s disease may trigger abnormal Th17 and Th1 immune responses

**DOI:** 10.1038/srep20401

**Published:** 2016-02-04

**Authors:** Zi Ye, Bolin Deng, Chaokui Wang, Dike Zhang, Aize Kijlstra, Peizeng Yang

**Affiliations:** 1 The First Affiliated Hospital of Chongqing Medical University, Chongqing Key Lab of Ophthalmology, Chongqing Eye Institute, Chongqing, P. R. China; 2 University Eye Clinic Maastricht, Maastricht, the Netherlands

## Abstract

Behcet’s disease (BD) is a chronic, systemic and recurrent inflammatory disease associated with hyperactive Th17 and Th1 immune responses. Recent studies have shown that B and T lymphocyte attenuator (BTLA) negatively regulates the immune response. In this study, we investigated whether BTLA activation could be exploited to inhibit the development of abnormal immune responses in BD patients. BTLA expression in PBMCs and CD4^+^ T cells was significantly decreased in active BD patients. Decreased BTLA level was associated with increased Th17 and Th1 responses. Activation of BTLA inhibited the abnormal Th17 and Th1 responses and IL-22 expression in both patients and controls. Addition of an agonistic anti-BTLA antibody remarkably inhibited DC-induced Th17 and Th1 cell responses, resulted in decreased production of the Th17 and Th1-related cytokines IL-1beta, IL-6, IL-23 and IL-12p70 and reduced CD40 expression in DCs. In conclusion, decreased BTLA expression in ocular BD may lead to inappropriate control of the Th17 and Th1 immune responses and DC functions. Therefore, BTLA may be involved in the development and recurrence of this disease. Agonistic agents of BTLA may represent a potential therapeutic approach for the treatment of BD and other inflammatory diseases mediated by abnormal Th17 and Th1 immune responses.

Uveitis is a great threat to vision worldwide. Behcet’s disease (BD) and Vogt-Koyanagi-Harada (VKH) syndrome are the two major sight-threatening uveitis entities in China. BD is a relapsing systemic autoinflammatory disorder characterized by recurrent uveitis, skin lesions, and oral and genital ulcerations and it is widespread in silk-road countries, such as China, Japan and Turkey^1^,^2^. VKH syndrome is an autoimmune disease directed against melanocyte antigens and is characterized by granulomatous panuveitis associated with multisystem involvement and frequently occurs in Asians and Native Americans^3^,^4^. Although the exact mechanism underlying the pathogenesis of these two uveitis entities is still not completely understood, an etiology involving certain infectious triggers has been suggested^5^,^6^. Numerous studies on autoimmune and autoinflammatory diseases have demonstrated a central role for T lymphocytes (especially Th17 and Th1 cells) in their pathogenesis. Previous studies demonstrated that hyperactive Th17 and Th1 immune responses and their associated cytokines were involved in the development of BD and VKH^7^,^8^,^9^,^10^,^11^,^12^. Suppressing abnormal Th17 and Th1 cell immune responses controls the inflammatory response in BD patients, VKH patients and experimental autoimmune uveitis (EAU), which is a typical animal model of human uveitis^13^,^14^,^15^,^16^.

The B and T lymphocyte attenuator (BTLA, also known as CD272) belongs to the CD28 family and has been identified as a coinhibitory molecule expressed on T and B lymphocytes as well as other immune cells, including dendritic cells (DCs), monocytes, natural killer cells and natural killer T cells^17^,^18^,^19^. BTLA is a membrane glycoprotein that contains two immunoreceptor tyrosine-based inhibition motifs (ITIMs). Engagement of BTLA by its ligand herpes virus entry mediator (HVEM) inhibits T-cell activation by reducing T-cell receptor (TCR) signaling phosphorylation^17^,^20^. Recent studies have demonstrated that BTLA^−/−^ mice were more susceptible to several inflammatory diseases compared with wild-type mice, including experimental autoimmune encephalomyelitis (EAE)^19^, airway inflammation^21^ and lipopolysaccharide (LPS)-induced endotoxic shock^22^. Treatment with anti-BTLA monoclonal antibodies (mAb) prevented Rag^−/−^ mice from experiencing the acceleration of T-cell-induced colitis^23^. These studies indicate that BTLA may control excessive inflammatory responses. However, whether BTLA expression is altered in clinical disease remains largely unknown and is the focus of the present study.

Given the excessive Th17 and Th1 immune responses in BD and VKH patients, we investigated whether BTLA was involved in the development of abnormal T-lymphocyte responses in patients with these two diseases. The results indicate that decreased BTLA expression was associated with increased Th17 and Th1 immune responses in BD but not VKH patients.

## Results

### BTLA expression is decreased on PBMCs and CD4^+^ T cells from active ocular BD patients

**Figure 1:**
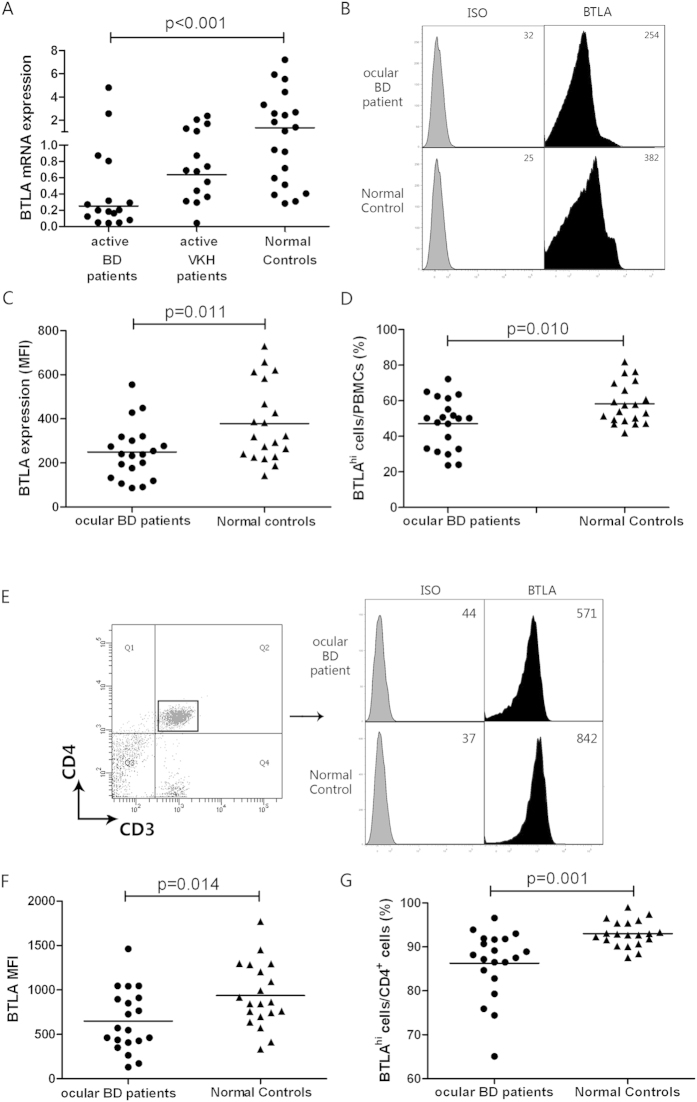
BTLA expression is decreased on PBMCs and CD4^+^ T cells from ocular BD patients. (**A**) Expression of BTLA mRNA in PBMCs from ocular BD patients (*n* = *16*), VKH patients (*n* = *15*) and normal controls (*n* = *20*). BTLA mRNA was evaluated by real-time PCR and normalized to beta-actin. BTLA protein expression was evaluated in PBMCs from ocular BD patients (*n* = *20*) and normal controls (*n* = *20*) by flow cytometry. (**B**) Histogram showing BTLA expression on PBMCs in a representative subject for each group is shown. (**C**) Median fluorescence intensity (MFI) of BTLA in PBMCs from both two groups. (**D**) Percentage of BTLA^hi^ cells in PBMCs from BD patients and healthy controls. BTLA protein expression was evaluated in CD3^+^CD4^+^ T cells from ocular BD patients (*n* = *20*) and normal controls (*n* = *20*) by flow cytometry. (**E**) Expression of BTLA on CD4^+^ T cells in a representative subject for each group is shown. (**F**) Data presented as MFI of BTLA in CD4^+^ T cells from both groups. (**G**) Frequency of BTLA^hi^ cells in the CD4^+^ T cell population from ocular BD patients and normal controls. One way ANOVA, Bonferroni post hoc test and Kruskal-Wallis H test for independent samples were used for statistical analyses.

Whether BTLA participates in the pathogenesis of uveitis is unknown. We therefore first compared the mRNA expression of BTLA in PBMCs from ocular BD patients, VKH patients and normal controls. The RT-PCR results showed that the BTLA mRNA expression was significantly decreased in BD patients with active ocular inflammation compared to the normal controls (*p* < 0.001); no differences were observed in the PBMCs from VKH patients and controls (Fig. 1A). The flow cytometry results also showed a lower protein expression of BTLA and a decreased percentage of BTLA^hi^ cells in the PBMCs from active ocular BD patients (*p* = 0.011, *p* = 0.010) (Fig. 1B–D).

To investigate the correlation between BTLA expression and the extraocular clinical manifestations, we compared the protein expression of BTLA in PBMCs from 20 active ocular BD patients with or without certain extraocular clinical features. No significant differences in BTLA expression was noted between ocular patients with genital ulcers, arthritis, skin lesions or positive skin allergy reactions and patients without these clinical findings (Supplementary Fig. 1).

To investigate the expression of BTLA on PBMC subsets, we compared the expression of BTLA in CD4^+^ T cells, CD8^+^ T cells, B cells and monocytes from ocular BD patients and normal controls by flow cytometry. Decreased protein expression of BTLA was observed in CD4^+^ T cells from ocular BD patients as compared with cells from the normal controls (*p* = 0.014) (Fig. 1E,F). In line with this result, a reduced percentage of CD4^+^BTLA^hi^ cells was also observed in the ocular BD patients compared with controls (*p* = 0.001)(Fig. 1G). No detectable differences in BTLA expression were observed in the CD8^+^ cells, B cells and monocytes from the ocular BD patients and the normal controls (Supplementary Fig. 2).

### Decreased BTLA expression is associated with increased Th17 and Th1 cell immune responses

**Figure 2:**
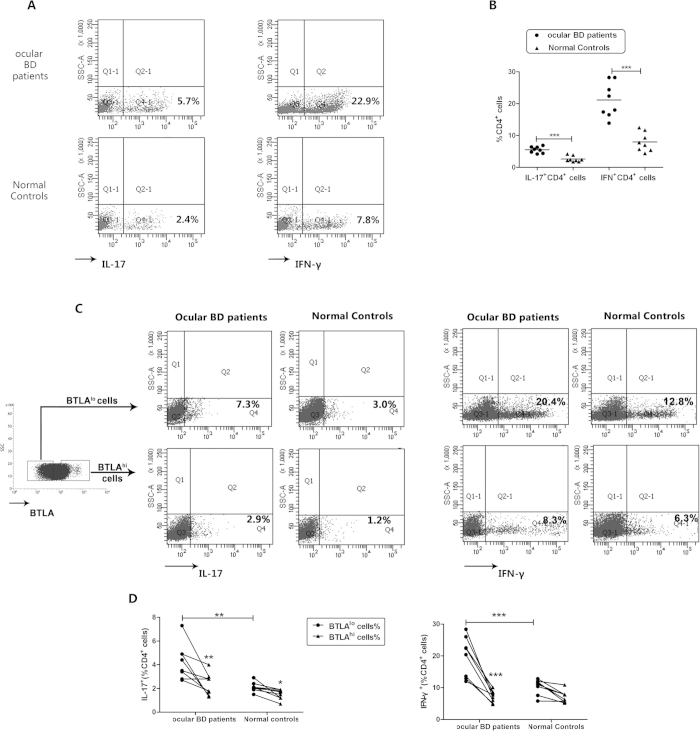
Decreased expression of BTLA is associated with increased Th17 and Th1 cell responses. Purified CD4^+^ T cells from ocular BD patients (*n* = *8*) and normal controls (*n* = *8*) were stimulated with anti-CD3/CD28 for 3 days. The cells were analyzed for intracellular expression of IL-17 and IFN-gamma by flow cytometry. (**A**) A scatter diagram of a representative subject for each group is presented. (**B**) Frequency of IL-17- and IFN-gamma-producing CD4^+^ T cells in patients and controls. Anti-CD3/CD28 stimulated CD4^+^BTLA^lo^ cells and CD4^+^BTLA^hi^ cells from patients (*n* = *8*) and controls (*n* = *8*) were analyzed for the expression of IL-17 and IFN-gamma by flow cytometry. (**C**) Scatter diagrams of a representative subject for each group are shown. (**D**) Frequency of IL-17- and IFN-gamma-producing in CD4^+^BTLA^hi^ cells and CD4^+^BTLA^lo^ cells. *p < 0.05, **p < 0.01, ***p < 0.001. One way ANOVA and Kruskal-Wallis H test for independent samples were used for statistical analyses.

Because recent studies indicated that CD4^+^ T cells (especially Th17 and Th1 cells) played a role in the inflammatory activity of BD^8^,^11^,^24^ and since a decreased expression of BTLA in CD4^+^ T cells was observed in ocular BD patients, we investigated the possible relationship between BTLA expression and the Th17 and Th1 cell responses in ocular BD patients and normal controls. We analyzed the Th17 and Th1 responses in CD4^+^ T cells from the ocular BD patients and normal controls by flow cytometry. Consistent with earlier observations^8^,^9^,^10^,^11^,^12^, a significantly increased IL-17 and IFN-gamma expression was observed in CD4^+^ T cells from the ocular BD patients compared with the normal controls (*p* < 0.001, *p* < 0.001) (Fig. 2A,B).

The CD4^+^ T cells from the ocular BD patients and normal controls were divided into two subsets based on BTLA expression and then stimulated with anti-CD3/anti-CD28 for 72 h. The frequencies of IL-17- and IFN-gamma-positive cells were compared among the CD4^+^BTLA^hi^ cells and CD4^+^BTLA^lo^ cells from the ocular BD patients and normal controls. CD4^+^BTLA^lo^ cells were more often IL-17- and IFN-gamma-positive compared than CD4^+^BTLA^hi^ cells in the ocular BD patients (*p* < 0.01, *p* < 0.001). The percentage of IL-17-positive cells was often increased in the CD4^+^BTLA^lo^ cell population compared with the CD4^+^BTLA^hi^ cell population in the normal controls (*p* < 0.05). The frequencies of IL-17- and IFN-gamma-positive cells were significantly increased in the CD4^+^BTLA^lo^ cells population from the ocular BD patients compared with the same cell population from the normal controls (*p* < 0.01, *p* < 0.001). There was no difference in the IL-17- and IFN-gamma-positive cell frequencies in CD4^+^BTLA^hi^ cells from BD patients and normal controls (Fig. 2C,D).

### Agonistic anti-BTLA antibody inhibits hyperactive Th17 and Th1 responses in both BD patients and normal controls

**Figure 3:**
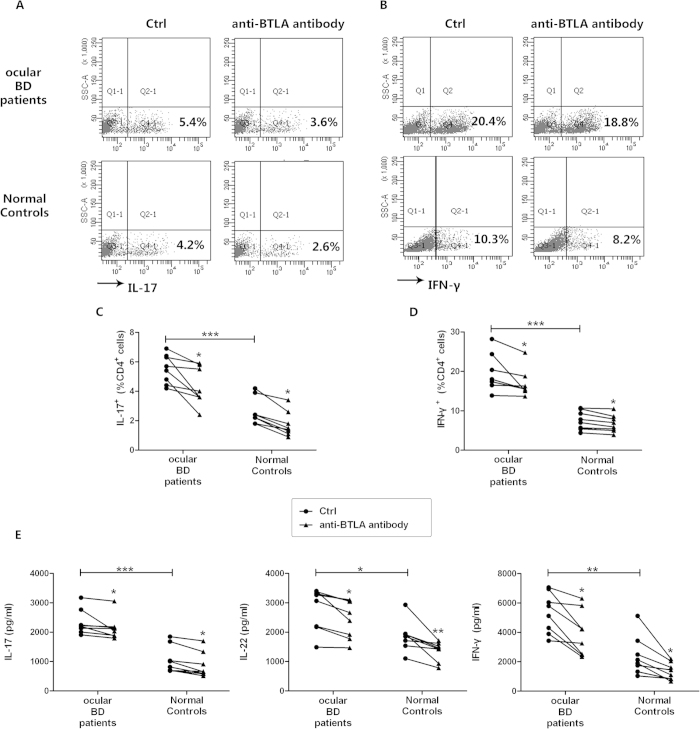
Agonistic anti-BTLA antibody inhibits overreacted Th17 and Th1 cell responses. Purified CD4^+^ T cells from ocular BD patients (*n* = *8*) and normal controls (*n* = *8*) were stimulated with anti-CD3/CD28 in the presence or absence of 1μg/ml agonistic anti-BTLA antibody for 3 days. (**A,B**) The cells were stimulated with PMA/ionomycin and analyzed for intracellular expression of IL-17 and IFN-gamma by flow cytometry. Scatter diagrams of a representative subject for each group are shown. (**C,D**) Frequency of IL-17- and IFN-gamma-producing CD4^+^ T cells. (**E**) The production of IL-17, IL-22 and IFN-gamma in the supernatants was determined by ELISA. *p < 0.05, **p < 0.01, ***p < 0.001. One way ANOVA and Wilcoxon test for paired samples, Mann-Whitney U test for independent samples were used for statistical analyses.

The aforementioned results showed that the decreased expression of BTLA in PBMCs and CD4^+^ T cells was associated with an increased Th17 and Th1 cell frequency in the ocular BD patients. To investigate the effect of BTLA on the Th17 and Th1 immune responses, an agonistic reagent for BTLA was used to stimulate CD4^+^ T cells from the ocular BD patients and normal controls for 72 h. Intracellular cytokine analysis revealed that the agonistic anti-BTLA antibody inhibited the frequency of IL-17- and IFN-gamma-producing CD4^+^ T cells in both the patients (*p* < 0.05, *p* < 0.05) and controls (*p* < 0.05, *p* < 0.05)(Fig. 3A–D). The ELISA results demonstrated that the IL-17, IL-22 (another important cytokine produced by Th17 cells^25^,^26^) and IFN-gamma levels were significantly increased in the ocular BD patients (*p* < 0.001, *p* < 0.05, *p* < 0.01). Furthermore, the agonistic anti-BTLA antibody inhibited the production of the above-mentioned cytokines in both patients (*p* < 0.05, *p* < 0.05, *p* < 0.05) and controls (*p* < 0.05, *p* < 0.01, *p* < 0.05)(Fig. 3E).

### BTLA suppresses DC-induced Th17 and Th1 cell responses in CD4^+^ T cells

**Figure 4:**
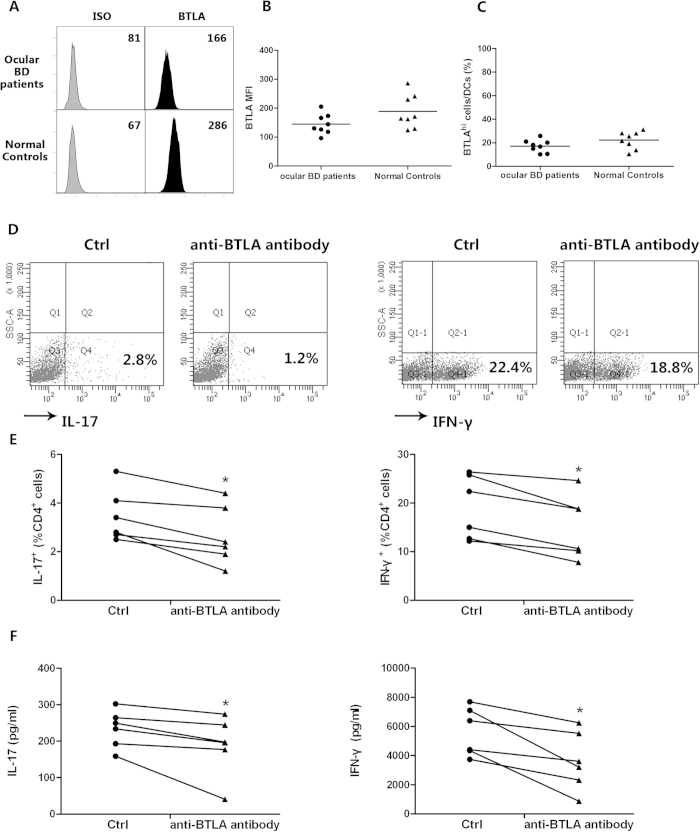
BTLA suppresses DC-induced Th17 and Th1 cell responses in CD4^+^ T cells. Monocytes from ocular BD patients (*n* = *8*) and normal controls (*n* = *8*) were stimulated with 100 ng/ml GM-CSF and 50 ng/ml IL-4 for 6 days and then stimulated with 100ng/ml LPS to generate DCs. (**A**–**C**) The expression of BTLA and percentage of BTLA^hi^ cells in DCs from ocular BD patients and healthy controls. Monocyte-derived DC from normal controls (*n* = *6*) were stimulated with or without agonistic anti-BTLA antibody and co-cultured with allogeneic CD4^+^ T cells for another 5 days. (**D**) The cells were stimulated with PMA/ionomycin and analyzed for intracellular expression of IL-17 and IFN-gamma by flow cytometry. Scatter diagrams of a representative subject for each group are shown. (**E**) Frequency of IL-17- and IFN-gamma-producing CD4^+^ T cells following BTLA stimulation. (**F**) The release of IL-17 and IFN-gamma in the culture supernatants was determined by ELISA. *p < 0.05, **p < 0.01, ***p < 0.001. One way ANOVA, Kruskal-Wallis H test for independent samples and Wilcoxon test for paired samples were used for statistical analyses.

As an important professional antigen presenting cell, DCs can induce the activation of T helper cell subsets^27^,^28^. To investigate whether BTLA activation of DCs had an effect on the development of Th17 and Th1 cells, we first compared the expression of BTLA in monocyte-derived DCs from the ocular BD patients and normal controls. The results showed that no statistically significant difference was observed between these two groups, which was in line with the results in monocytes (Fig. 4A–C). Then we co-cultured the agonistic anti-BTLA antibody stimulated DCs or control DC with CD4^+^ T cells for 5 days. The results showed that the frequencies of IL-17- and IFN-gamma-producing cells were significantly decreased in CD4^+^ T cells co-cultured with agonistic anti-BTLA antibody stimulated DCs as compared with co-culture with control DCs (*p* < 0.05, *p* < 0.05)(Fig. 4D–E). The agonistic anti-BTLA antibody also inhibited the release of IL-17 and IFN-gamma in the culture supernatants (*p* < 0.05, *p* < 0.05)(Fig. 4F).

### BTLA diminishes the expression of co-stimulatory molecules and Th17- and Th1-inducing cytokines by DCs in both BD patients and normal controls

**Figure 5:**
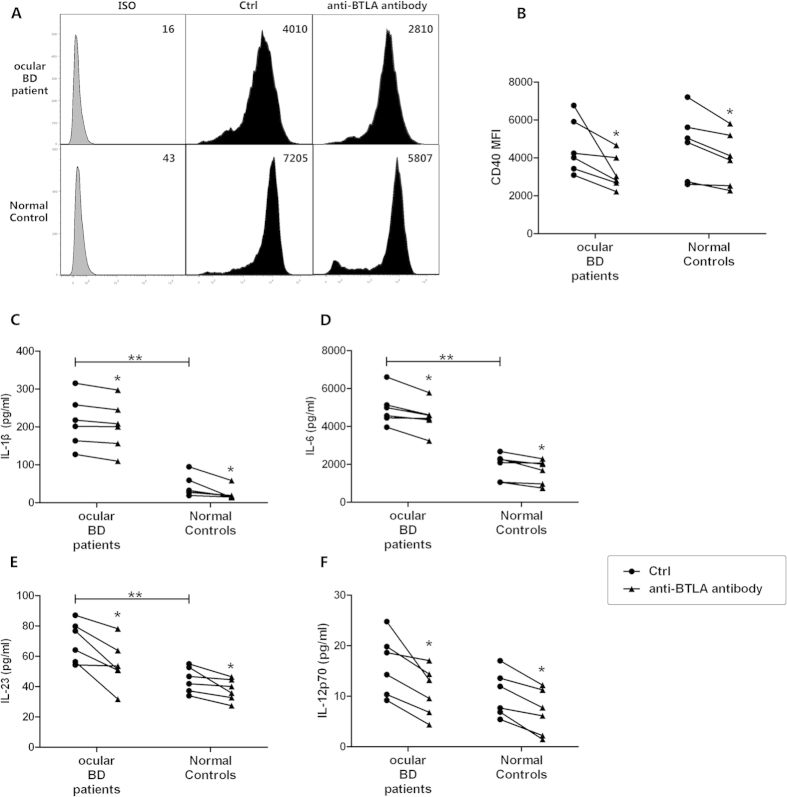
BTLA diminishes the expression of co-stimulatory molecules and Th17-/Th1-inducing cytokines by DCs both in BD patients and normal controls. Monocytes from ocular BD patients (*n* = *6*) and normal controls (*n* = *6*) were stimulated with 100 ng/ml GM-CSF and 50 ng/ml IL-4 for 6 days to generate DCs. The cells were then stimulated with LPS or LPS plus 1μg/ml agonistic anti-BTLA antibody for 24h. Cells were harvested for co-stimulatory molecule analysis by flow cytometry. (**A**) Histograms of CD40 expression of a representative subject for each group are shown. (**B**) Data presented as MFI of CD40 in DCs. (**C–F**) The expression of IL-1beta, IL-6, IL-23 and IL-12p70 in the supernatants was determined by ELISA. *p < 0.05, **p < 0.01, ***p < 0.001. One way ANOVA and Wilcoxon test for paired samples were used for statistical analyses.

The expression of co-stimulatory molecules is essential for the antigen presentation capability of DCs and the induction of the T helper cell response^29^,^30^,^31^. To investigate whether the inhibitory effect of BTLA-stimulated DCs on the development of Th17 and Th1 cells was mediated by modulating the expression of co-stimulatory molecules, agonistic anti-BTLA antibody-treated DCs and control DCs from the ocular BD patients and normal controls were analyzed for CD83, CD80, CD86, CD40 and HLA-DR expression by flow cytometry. The results indicated that BTLA stimulation only down-regulated the expression of CD40 on DCs in both the ocular BD patients and normal controls (*p* < 0.05, *p* < 0.05)(Fig. 5A,B). However, BTLA had no effect on the other four molecules assessed (Supplementary Fig. 3).

Next, we investigated the effect of BTLA on the production of cytokines by DCs that facilitated Th17 and Th1 immune responses. The expression of the Th-17-inducing cytokines IL-1beta, IL-6 and IL-23 was significantly increased in ocular BD patients as compared with normal controls (*p* < 0.01, *p* < 0.01, *p* < 0.01), and BTLA stimulation decreased their production by DCs in both patients (*p* < 0.05, *p* < 0.05, *p* < 0.05) and controls (*p* < 0.05, *p* < 0.05, *p* < 0.05)(Fig. 5C–E). The expression of the Th1-inducing cytokine IL-12p70 was also inhibited following BTLA stimulation (*p* < 0.05, *p* < 0.05)(Fig. 5F). No difference in IL-12p70 expression was noted between the ocular BD patients and normal controls.

## Discussion

In this study, we observed a decreased BTLA expression in PBMCs and CD4^+^ T cells from ocular BD patients as compared with normal controls but no differences were noted for VKH patients. Additionally, no differences were observed in BTLA expression in BD patients with or without extraocular manifestations. These findings suggest that BTLA may contribute to the pathogenesis of uveitis in BD but not in VKH. This may be due to the different immunopathology of these two diseases. BD is considered an autoinflammatory disease, whereas VKH is seen as an autoimmune disease directed against melanocyte antigens. To avoid the effect of immunosuppressive agents on BTLA expression, patients treated with immunosuppressive agents were excluded from this study. However, it would be interesting to address the influence of treatment on BTLA expression in future studies. Most studies on BTLA expression in human disease have focused on virus infection^32^,^33^,^34^,^35^. Earlier studies investigating the expression of BTLA on CD4^+^ T cells, CD8^+^ T cells and B cells showed that it was significantly decreased in patients with HIV-1 infection as compared with normal controls^32^,^35^. To the best of our knowledge, no studies have been reported on BTLA expression during inflammatory diseases in humans, and to date, only studies in mice have been published^19^,^21^.

Various studies indicate that hyperactive Th17 and Th1 immune responses play a vital role in the development of BD^8^,^10^,^11^. Our results confirmed these findings and revealed markedly increased frequencies of IL-17-producing and IFN-gamma-producing CD4^+^ T cells in ocular BD patients. To further investigate whether decreased BTLA might influence the Th17 and Th1 immune responses, a number of experiments were performed. First, we compared the frequency of IL-17 and IFN-gamma expressing CD4^+^BTLA^lo^ cells and CD4^+^BTLA^hi^ cells, and found a significantly increased frequency of IL-17 and IFN-gamma in CD4^+^BTLA^lo^ cells. We next stimulated BTLA by the addition of the agonistic anti-BTLA antibody to CD4^+^ T cell cultures and found that BTLA activation inhibited the production of IL-17, IL-22 and IFN-gamma. Intracellular staining confirmed the down-regulatory effect of BTLA on the frequencies of IL-17- and IFN-producing CD4^+^ T cells. Our findings concerning IFN-gamma are consistent with data from previous studies in mice demonstrating that BTLA activation inhibited the production of IFN-gamma^36^,^37^. Recent studies showed that CD27^−^ gamma delta T cells from BTLA^−/−^ mice produced more IL-17 as compared with wild type mice^38^. Both our data and the mouse studies suggest that BTLA may control Th17 and Th1 immune responses and reduced expression of this molecule may play a role in the development of ocular BD. Notably, a complex relationship exists among the Th17, Th1 and Treg cells. These three T cell subsets can convert into one another under different inflammatory conditions^10^,^39^. An increased Th17/Treg ratio was observed in the cerebrospinal fluid and PBMCs of patients with BD and was proposed to correlate with disease activity^40^,^41^. These studies indicated that Treg cells also played a role in the pathogenesis of BD. Therefore, whether BTLA influences the quantity and function of Treg cells deserves further study.

DCs act as a bridge that contributes to both the innate and adaptive immune responses. Interaction of the pattern recognition receptors (such as Toll-like receptors) expressed on DCs with microbial antigens results in the induction of T cell-mediated immune responses^27^,^28^. Recent studies showed that BTLA not only played a negative regulatory role for T and B cells but also suppressed the LPS-induced TLR4 signaling in DCs^22^. In BTLA-deficient mice, the number of DCs was significantly increased in the spleen as compared with the wild-type mice^42^. In view of the interaction between BTLA and DCs in mice, we also studied the effect of this molecule on the function of human DCs. As a model for DCs, we chose to use *in vitro* monocyte-derived DCs and used an agonistic anti-BTLA antibody to stimulate BTLA. The results indicate that the frequencies of Th17 and Th1 cells were significantly decreased in CD4^+^ T cells co-cultured with the agonistic anti-BTLA antibody-stimulated DCs. Release of IL-17 and IFN-gamma in these co-culture supernatants was also inhibited following BTLA stimulation. Taken together, these results provided evidence that BTLA played a negative regulatory effect on Th17 and Th1 cell immune responses in a manner that was possibly mediated by DCs. DCs regulate the differentiation of Th cell subsets, including Th17 and Th1 cells, by secreting different cytokines. The production of IL-1beta, IL-6 and IL-23 by DCs is capable of inducing the development of Th17 cells, whereas IL-12p70 is a crucial factor for the differentiation of Th1 cells^43^,^44^,^45^. We examined the effect of BTLA on the production of these Th17 and Th1 cell-related cytokines by DCs from both ocular BD patients and normal controls. In agreement with the previous studies^46^,^47^, IL-1beta, IL-6 and IL-23 expression was significantly increased in active ocular BD patients, thus indicating the presence of hyperactive DCs in these patients. Agonistic anti-BTLA antibody-stimulation of BTLA inhibited the expression of these three Th17-related cytokines in both the ocular BD patients and normal controls as well as IL-12p70 production. Recent studies reported that BTLA^−/−^ DCs produced large amounts of IL-12 and TNF-alpha in the murine LPS-induced endotoxin shock model^22^. In addition to cytokine secretion, the cell-cell interaction between DCs and T cells is also important for the Th cell response. The expression of co-stimulatory molecules such as MHC-II, CD86, CD80, CD83 and CD40 expressed on DCs promote TRC signaling and lead to T cell activation and differentiation^31^,^48^. Stimulation of BTLA in DC cultures resulted in the significant inhibition of CD40 expression on DCs from both the ocular BD patients and normal controls. We failed to find an effect of BTLA on the expression of CD86, CD83, CD80 and HLA-DR. Several studies investigating interaction of CD40 and its ligand CD40L showed that this interaction played an important role in Th17 and Th1 cell responses^49^,^50^,^51^. Moreover, Iezzi *et al*. found that CD40^−/−^ DCs failed to induce Th17 development *in vitro*^50^. Our finding that BTLA activation regulated the expression of CD40 on DCs might explain the inhibitory effect of BTLA on the generation of Th17 and Th1 cells. Further studies are required to exactly determine the precise role of BTLA in the control of the Th17 and Th1 immune responses and to clarify whether this regulation is mediated via DCs.

We and others have examined the function of BTLA by adding an agonistic anti-BTLA antibody to T lymphocyte cultures^37^,^52^. These agonistic antibodies are considered to crosslink BTLA molecules on the cell surface, thereby sending an “off” signal to the T cell^37^. Confirmation of the role of BTLA by using other types of antibodies that would block the effect of BTLA have not yet been identified and would strengthen the proposed working mechanism of BTLA.

In conclusion, our study showed a decreased expression of BTLA in ocular BD and we provide preliminary data suggesting that this decrease might affect the control of the Th1 and Th17 responses thus contributing to the development of this disease.

## Materials and Methods

### Subjects

A total of 68 Chinese BD patients with active ocular inflammation (39 men and 29 women, average 39.3 years), 15 Chinese VKH patients with active ocular inflammation (9 men and 6 women, average 32.5 years) and 79 age- and sex-matched normal controls were enrolled in this study. The diagnoses of BD and VKH syndrome were made according to the diagnostic criteria designed by the International Study Group for Behcet’s disease and the diagnostic criteria revised for VKH by an international committee on nomenclature, respectively^53^,^54^. BD patients with active ocular inflammation presented the following ocular manifestations: floating cells in the anterior chamber or vitreous (100%), retinal vasculitis (100%) and hypopyon (28%). Recurrent oral ulcers were noted in all patients. In addition, genital ulcers, arthritis, skin lesions and positive skin allergy reactions were found in 49%, 26%, 46% and 15% of the patients, respectively. All VKH patients with active ocular inflammation presented cells in the anterior chamber, mutton-fat keratic precipitates, sunset glow fundus and Dalen-Fuchs nodules. The extraocular findings in VKH patients included poliosis (33%), alopecia (27%), vitiligo (27%), tinnitus (40%) and dysacusis (40%). All of the VKH patients and 64 out of the 68 BD patients had not yet received treatment with immunosuppressive drugs and the remaining 4 BD patients had received a low dose of oral prednisone (<10 mg/d) but had stopped using the drugs approximately one week prior to the blood sampling for the current study. All procedures followed the tenets of the Declaration of Helsinki and were approved by the Clinical Ethical Research Committee of Chongqing Medical University. All subjects provided written informed consent for the study.

### Cell isolation and culture

Peripheral blood mononuclear cells (PBMCs) were isolated from fresh heparinized blood by Ficoll-Hypaque density gradient centrifugation. CD4^+^ T cells and CD14^+^ monocytes were purified from PBMCs from active ocular BD patients and the normal controls using CD4 and CD14 mAb-conjugated magnetic microbeads (Miltenyi Biotec, Bergisch Gladbach, Germany) according to the manufacturer’s instruction. The purity of the CD4^+^ T cells and CD14^+^ monocytes was >90%.

CD4^+^ T cells were stained with anti-human BTLA (CD272)-PE (Biolegend, San Diego, CA) for 30 min and sorted into BTLA^hi^ cells and BTLA^lo^ cells using a BD FACs Aria™ II cell sorter. Then, the cells were cultured in RPMI 1640 medium containing 10% fetal bovine serum (FBS) and 1% penicillin/streptomycin. The cells were stimulated with 1μg/ml anti-CD3 and 1μg/ml anti-CD28 (eBioscience, San Diego, CA) for 72 h to detect interleukin (IL)-17 and interferon (IFN)-gamma by flow cytometry. The CD4^+^ T cells were also stimulated with anti-CD3 and anti-CD28 for 72 h to detect the Th17 and Th1 response or stimulated with anti-CD3 and anti-CD28 in the presence or absence of 1μg/ml agonistic anti-BTLA antibody (Clone MIH26, Biolegend)^37^,^52^ for 72 h to detect the effect of BTLA on Th17 and Th1 immune responses.

DCs were generated from CD14^+^ monocytes by stimulation with 100 ng/ml human GM-CSF and 50 ng/ml rIL-4 (both from R&D systems, Minneapolis, MN) for 72 h. Then, half of the culture medium (including the recombinant cytokines described above) was refreshed. Monocyte-derived DCs were stimulated with 100 ng/ml LPS (Sigma-Aldrich, St Louis, Mo) for 24 h to detect the expression of BTLA Additionally, the DCs were stimulated with 100 ng/ml LPS in the presence or absence of 1 μg/ml agonistic anti-BTLA antibody for 24 h to measure the effect of BTLA on co-stimulatory molecule expression and cytokine production by DCs.

CD4^+^ T cells separated from the normal controls were co-cultured with LPS -stimulated monocyte-derived DCs with or without 1 μg/ml of an agonistic anti-BTLA antibody for 5 days. The ratio of CD4^+^ T cells to monocyte-derived DCs was 5:1.

### Real-time quantitative polymerase chain reaction (RT-PCR)

Total RNA was isolated from PBMCs using the RNAeasy Mini Kit (Qiagen, Valencia, CA) according to the manufacturer’s instructions. The superscript III Reverse Transcriptase system (Invitrogen, Carlsbad, CA) was used to synthesize cDNA. Real-time analysis of BTLA and beta-actin mRNA was performed using SYBR Premix Ex Taq^TM^ II (TAKARA, Dalian, China) on the ABI 7500 Real-Time PCR System (Applied Biosystems) according to the manufacturer’s instructions. The primers for BTLA were purchased from Qiagen. The primers used for beta-actin were described elsewhere^55^. Data were normalized to the expression of beta-actin using the 2^−ΔΔCT^ method as described previously^56^.

### Flow cytometry

PBMCs from active ocular BD patients and normal controls were incubated with anti-human BTLA (CD272)-PE (Biolegend), anti-human CD3-(PerCP)-Cy5.5, anti-human CD4-APC, anti-human CD8-FITC, anti-human CD14-FITC (ebioscience) and anti-human CD19-APC (Miltenyi Biotec) at 4 °C for 30 min according to the manufacturer’s instructions to detect BTLA expression in PBMCs, CD4^+^ T cells, CD8^+^ T cells, monocytes and B cells. DCs were also incubated with anti-human BTLA (CD272)-PE at 4 °C for 30 min to detect BTLA expression on DCs.

Cultured CD4^+^ T cells, CD4^+^BTLA^hi^ cells, CD4^+^BTLA^lo^ cells and co-cultured cells were stimulated with 50 ng/ml PMA and 1 μg/ml ionomycin (both from Sigma-Aldrich) for 1 h at 37 °C and then incubated with 10 μg/ml brefeldin A(Sigma-Aldrich) for an additional 4 h. To detect the IL-17 and IFN-gamma production by the BTLA^hi^ and BTLA^lo^ cells, CD4^+^BTLA^hi^ cells and CD4^+^BTLA^lo^ cells were fixed and permeabilized using the eBioscience Cytofix/Cytoperm kit. The cells were incubated with anti-human IL-17A-APC and anti-human IFN-gamma-FITC (both from eBioscience) for an additional 30 min at 4 °C. To detect the effect of the agonistic anti-BTLA antibody on the CD4^+^ T cells and DC-induced T cells, anti-BTLA antibody stimulated CD4^+^ T cells and co-cultured cells were performed as describe above.

Cultured monocyte-derived DCs were incubated with anti-human CD86-PE, anti-human CD80-FITC, anti-human CD40-FITC, anti-human CD83-FITC and anti-human HLA-DR-PE/Cy5 (all from eBioscience) at 4 °C for 30 min to detect the effect of BTLA on co-stimulatory molecule expression on DCs.

The flow cytometry data were measured and statistically analyzed as the median fluorescence intensity (MFI) or percentage of stained cells.

### Enzyme-linked immunosorbent assay (ELISA)

The human Duoset ELISA kits (R&D Systems) were used to measure the concentrations of IL-17, IL-22 and IFN-gamma in the culture supernatants of CD4^+^ T cells and the co-culture supernatants and the concentrations of IL-6 and IL-1beta in the DC cultures. The concentrations of IL-12p70 and IL-23 in the culture supernatants of DCs were measured with the human IL-12(p70) high sensitivity ELISA kit and the human IL-23 ELISA Ready-SET-GO! kit, respectively (both from eBioscience). All of the procedures followed the manufacturers’ instructions.

### Statistical Analysis

Data are shown as dot plots. The one way ANOVA, independent sample *t*-test, Bonferroni correction, Mann-Whitney test, Wilcoxon test and Kruskal-Wallis H test were applied using the SPSS 17.0 software (SPSS Inc, Chicago, Illinois, USA). A p-value less than 0.05 was considered statistically significant.

## Additional Information

**How to cite this article**: Ye, Z. *et al*. Decreased B and T lymphocyte attenuator in Behcet's disease may trigger abnormal Th17 and Th1 immune responses. *Sci. Rep.* **6**, 20401; doi: 10.1038/srep20401 (2016).

## Supplementary Material

Supplementary Figures
