# Supplementary material for: Decreased B and T lymphocyte attenuator in Behcet’s disease may trigger abnormal Th17 and Th1 immune responses
Source: Sci Rep. 2016 Feb 4;6:20401. doi: 10.1038/srep20401 (PMC4740741; doi:10.1038/srep20401)
Supplement: Supplementary Figures [file srep20401-s1.doc]

**Decreased B and T lymphocyte attenuator in Behcet’s disease may trigger abnormal Th17 and Th1 immune responses**

**Zi Ye^1^,** **Bolin Deng^1^,** **Chaokui Wang^1^,** **Dike Zhang^1^,** **Aize Kijlstra^2^,** **PeizengYang^1,*^**

**^1^** The First Affiliated Hospital of Chongqing Medical University, Chongqing Key Lab of Ophthalmology, Chongqing Eye Institute, Chongqing, P. R. China

^2^ University Eye Clinic Maastricht, Maastricht, the Netherlands

**Corresponding author:** Prof. Dr. Peizeng Yang, MD, PhD

The First Affiliated Hospital of Chongqing Medical University, Youyi Road 1, Chongqing, 400016, P. R. China

Fax & Phone: 0086-23-89012851

E-mail address: [peizengycmu@126.com](mailto:peizengycmu@126.com)

**Supplementary Figure 1:**


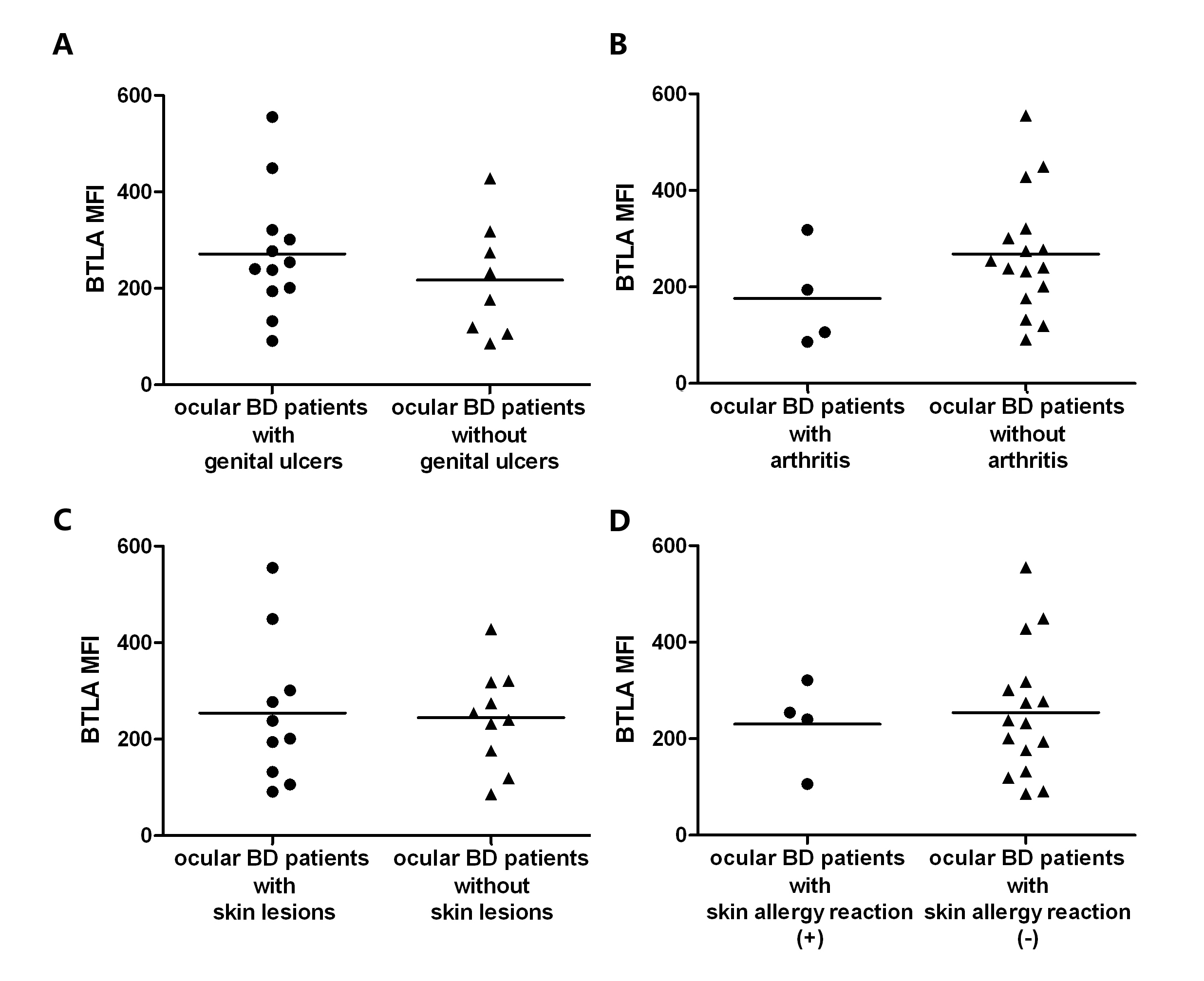


**Legend:** **The expression of BTLA expression in PBMCs from the ocular BD patients with extraocular clinical manifestations.** PBMCs were obtained from 20 ocular BD patients with or without several extraocular clinical manifestations. BTLA expression was analysed by flow cytometry. (A) The expression of BTLA in the PBMCs from ocular BD patients with (n=12) or without (n=8) genital ulcers. (B) The expression of BTLA in the PBMCs from ocular BD patients with (n=4) or without (n=16) arthritis. (C) The expression of BTLA in the PBMCs from ocular BD patients with (n=10) or without (n=10) skin lesions. (D) The expression of BTLA in the PBMCs from ocular BD patients with (n=4) or without (n=16) positive skin pathergy reaction. One way ANOVA, Independent samples *t* test for independent samples were used for statistical analyses.

**Supplementary Figure 2:**

**
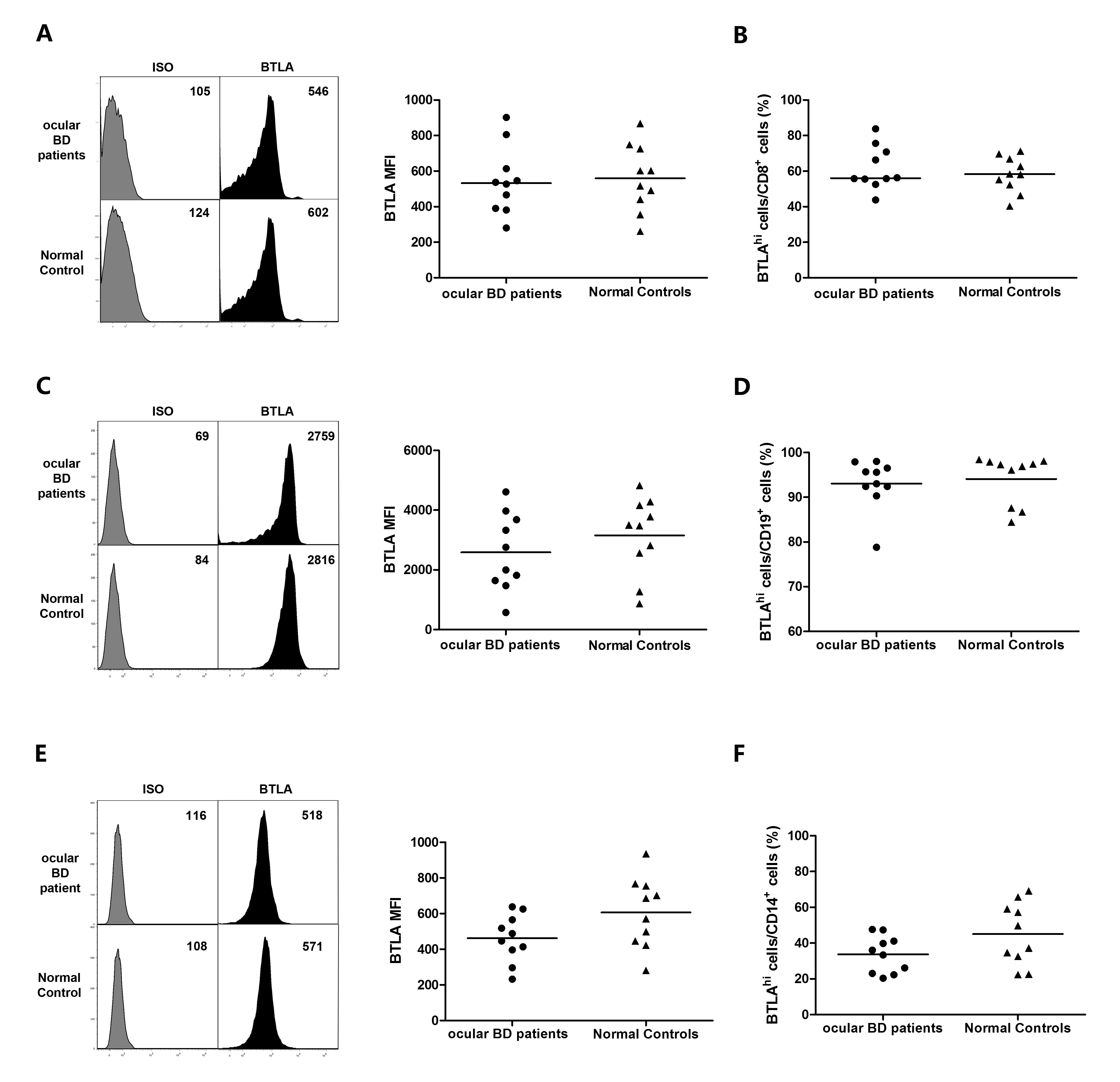
**

**Legend: The expression of BTLA expression in PBMC subsets from the ocular BD patients and normal controls.** PBMCs were obtained from ocular BD patients *(n=10)* and normal controls *(n=10)*. (A-B) The expression of BTLA and percentage of BTLA^hi^ cells in CD8^+^ T cells from ocular BD patients and healthy controls. (C-D) The expression of BTLA and percentage of BTLA^hi^ cells in B cells from ocular BD patients and healthy controls. (E-F) The expression of BTLA and percentage of BTLA^hi^ cells in monocytes from ocular BD patients and healthy controls. One way ANOVA, Independent samples *t* test and Kruskal-Wallis H test for independent samples were used for statistical analyses.

**Supplementary Figure 3:**


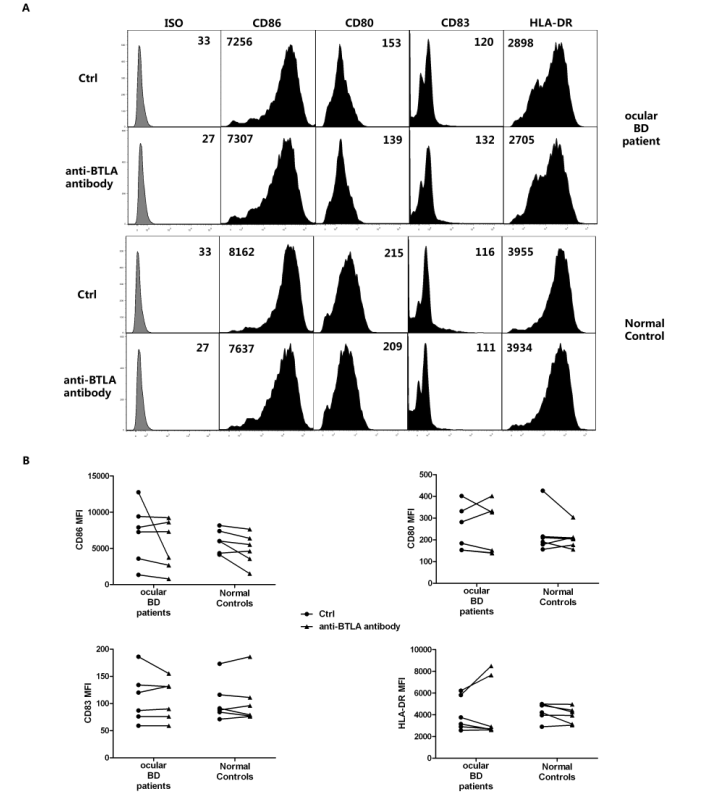


**Legend:** **The effect of agonistic anti-BTLA antibody on the expression of co-stimulatory molecular in DCs from ocular BD patients and normal controls.** Monocytes from ocular BD patients *(n=6)* and normal controls *(n=6)* were stimulated with 100 ng/ml GM-CSF and 50 ng/ml IL-4 for 6 days to generate DCs. The cells were then stimulated with LPS or LPS plus 1μg/ml agonistic anti-BTLA antibody for 24h. Cells were harvested for co-stimulatory molecule analysis by flow cytometry. (A) Histograms for CD86, CD80, CD83 and HLA-DR expression of a representative subject of each group are shown. (B) Data for the expression of the four co-stimulatory molecules are shown as MFI. One way ANOVA and Wilcoxon test for paired samples were used for statistical analyses.
